# A Meta-Analysis of the Motion Function through the Therapy of Spinal Cord Injury with Intravenous Transplantation of Bone Marrow Mesenchymal Stem Cells in Rats

**DOI:** 10.1371/journal.pone.0093487

**Published:** 2014-04-01

**Authors:** Duo Zhang, Xijing He

**Affiliations:** Department of Orthopaedics, Second Affiliated Hospital of Medical College of Xi'an Jiaotong University, Xi'an, Shaanxi Province, People's Republic of China; Indian Institute of Toxicology Research, India

## Abstract

**Background:**

To compare the efficacy of the therapy of spinal cord injury with intravenous transplantation of bone marrow mesenchymal stem cells (BMSCs) by Meta-analysis.

**Methods:**

Studies of the BBB scores after intravenous transplantation of BMSCs were searched out from Pubmed, SCI, Cochrane Library, Chinese journal full-text database, China Biology Medicinedisc and Wanfang data-base and analyzed by Review Manager 5.2.5.

**Results:**

Nine randomized controlled animal trials were selected with 235 rats enrolled. The studies are divided to different subgroups by different models of SCI and different time to transplantion. The results of Meta-analysis in different subgroups both indicated that the rats of experimental group (BMSCs group) got better BBB scores than control group at 1, 3 and over 5 weeks after intravenous transplantation of BMSCs with significant differences. The heterogeneity between impacted injury model and oppressed injury model subgroups decreased with the passage of time (I^2^ = 75.8%, 39.7%, 0%). No heterogeneity was found between 3 d and 7 d subgroups.

**Conclusion:**

The intravenous transplantation of BMSCs is an efficient way to cure spinal cord injury, which can improve the motor function of rats. The therapeutic window is wide.

## Introduction

With the development of economy and society, more and more cases of spinal cord injury (SCI) caused by jobs and traffic accidents have happened in recent years. Because of no definitely effective cure, SCI is a huge burden to the patients and the relatives. As a consequence, the SCI causes a mass of social problems. Therefore, it is necessary to find better methods to cure.

At the moment, the methods applied in clinical are:(1)surgery: relieve the oppression, dispel the hydroncus, improve the local microcirculation;(1)drugs: glucocorticoids, lithium, neuroprotective agents and so on;(3)functional training and neurological rehabilitation [1]. Recent studies show that cell transplantation promote nerve regeneration. Bone marrow mesenchymal stem cells (BMSCs) are good seed cells for transplantation and concerned by more and more researchers because of the unique properties. It has been proved that transplantation injected in local injury position with BMSCs can repair the injured spinal cord and improve the neural function [2]–[4]. However, the application of local transplantation is limited due to the operation, is complicated and easily causes secondary injury. There are some experiments indicate that intravenous transplantation of BMSCs has good effects on SCI [5].

To evaluate the locomotor recovery with animal models of spinal cord injury, BBB scale which is a sensitive and reliability of locomotor rating scale and set up by Basso, Beattie and Bresnahan is widely used [6]–[9]. BBB scale is estimated by observing the movements of lower limbs and joints of rats in open field. The full scores of BBB rating scale are 21 points which means normal function. The less score the rats get, the worse function they have [6].

This systematic review and Meta-analysis of BBB score in SCI rats through the comparison between the intravenous transplantation group and the control group is expected to offer academic support for cure of SCI.

## Materials and Methods

### 1. Search strategy

Electronic databases included PubMed, Science Citation Index, Cochrane Library and CJFD were searched to retrieve related studies published between 2003 and 2013 with the Medical Subject Heading (MeSH) keywords “intravenous transplantation”, “bone marrow mesenchymal stem cells”, “transplantation” and “spinal cord injury”. The language was not restricted.

### 2. Inclusion criteria

The articles were considered eligible if the studies met the following inclusion criteria: 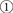
randomized controlled animal trials; 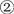
the research animals are SCI rats; 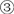
contained at least two groups: with and without intravenous transplantation of BMSCs; 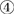
the results included at least BBB score; 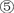
the control groups got the same model operation as the experiment groups but not injected with BMSCs.

### 3. Exclusion criteria

The articles were excluded if the studies met one of the following exclusion criteria: 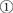
unable to get the full text; 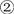
the author is same with another study; 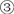
combined with other interventions; 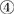
randomized controlled animal trial of low quality; 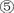
review.

### 4. Data extraction

The data was extracted independently by two reviewers and was rechecked after the extraction through reading the headlines, abstracts and the full text if necessary according to the inclusion and exclusion criteria. Any disagreement regarding eligibility during the extraction was discussed and resolved.

### 5. Assessment of methodology quality

The quality of the included studies was assessed according to Cochrane Handbook for Systematic Reviews of Interventions version 5.1.0. There are 6 items: 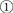
random sequence generation; 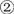
allocation concealment; 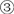
blinding of outcome assessment; 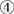
incomplete outcome data; 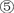
selective reporting; 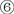
other bias. Every study was assessed by 2 independent researchers and the judgment of every item was low risk, unclear or high risk. Any disagreement regarding eligibility during the extraction was discussed and resolved.

### 6. Statistical analysis

The Meta-analysis was conducted using the RevMan software package (version 5.2.5; the Cochrane collaboration). For continuous variables, the weighted mean difference (WMD) were measured with the 95% CIs. WMDs were considered statistically significant at the P<0.05 level. Statistical heterogeneity among studies and subgroups was evaluated with the χ^2^ and I^2^ texts. Both a fixed-effects model and a random-effects model were used to obtain summary WMDs. The fixed-effects model was employed with the absence of heterogeneity, otherwise the random-effects model was employed. The following subgroup analyses were performed: (1)the outcomes of interest between different ways to make SCI models: strike or oppress. Strike models are set up with weight-drop, including Allen's weight-dropping model [10], improved Allen's weight-dropping models, NYU Impactor model and so on. Oppress models are set up with pressure, including clip compression [11]. (2)the outcomes of interest between transplantation after SCI 3 days and 7 days. A subgroup analysis was adopted to analyze the source of heterogeneity.

## Results

### 1. Description of studies

**Figure 1:**
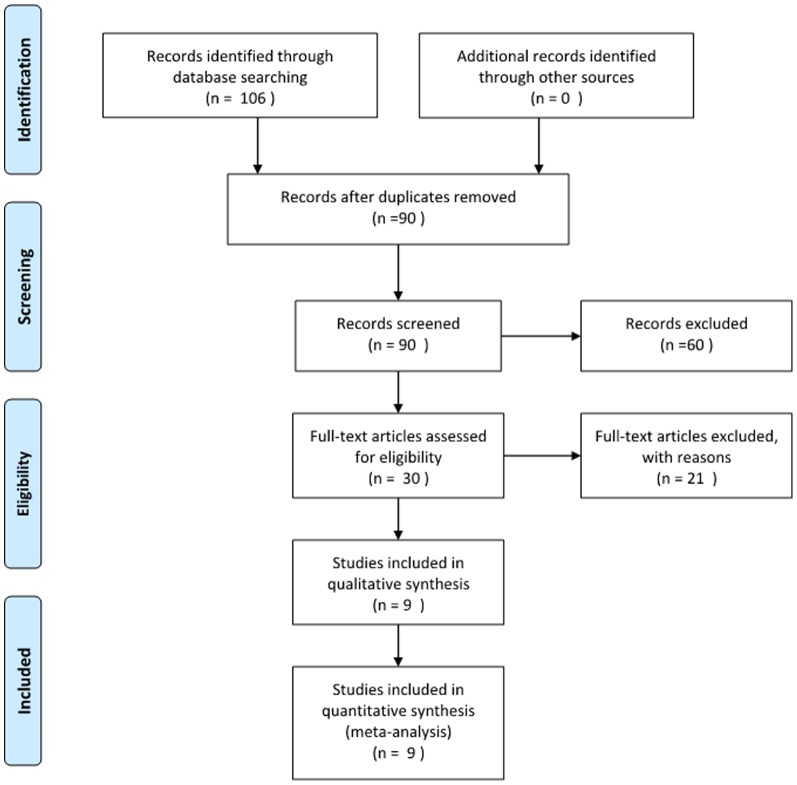
The process of identifying relevant srufies is summarized.

A total of 106 articles were initially identified after literature search by computer and hand. 97 studies were not up to the inclusion criteria through reading the titles, abstracts and the full text if necessary. A final total of 9 studies [1], [12]–[19] published from 2006 to 2012 were included in this meta-analysis (Figure 1). The SCI model applied by 7 studies was the impacted injury model, the others was the oppressed injury model. The BMSCs were transplanted 3 days after SCI in 5 studies and 7 days after SCI in 4 studies. (Table 1)

**Table 1:** Description of studies.

Author and Year	SCI Model	Time for transplantation (after SCI)	Cells count	Time to evaluate BBB score							
				1 w	2 w	3 w	4 w	5 w	6 w	7 w	>7 w
Feng DX 2006 [12]	II	7 d	2×10^6^	√	√	√	√	√	√		
Kang DZ 2006 [13]	II	3 d	1×10^6^	√		√		√			
Chen D 2007 [14]	II	7 d	1×10^5^	√	√	√					
Jing WL 2008 [15]	II	7 d	1×10^6^	√	√		√		√		
Xu XL 2009 [1]	OI	3 d	1×10^6^	√		√					√
Yu CS 2011 [16]	OI	7 d	1×10^6^	√		√				√	√
Zhao XZ 2011 [17]	II	3 d	1?5×10^6^	√	√	√					
Chen SQ 2012 [18]	II	3 d	1×10^6^	√		√		√			
Li BK 2012 [19]	II	7 d	1×10^6^	√		√				√	

“II” = impacted injury, the SCI model made by impacted injury”, “OI” = oppressed injury, the SCI model made by oppressed injury”, “d” = “days, “w” = “week”.

### 2. Risk of bias in included studies

**Figure 2:**
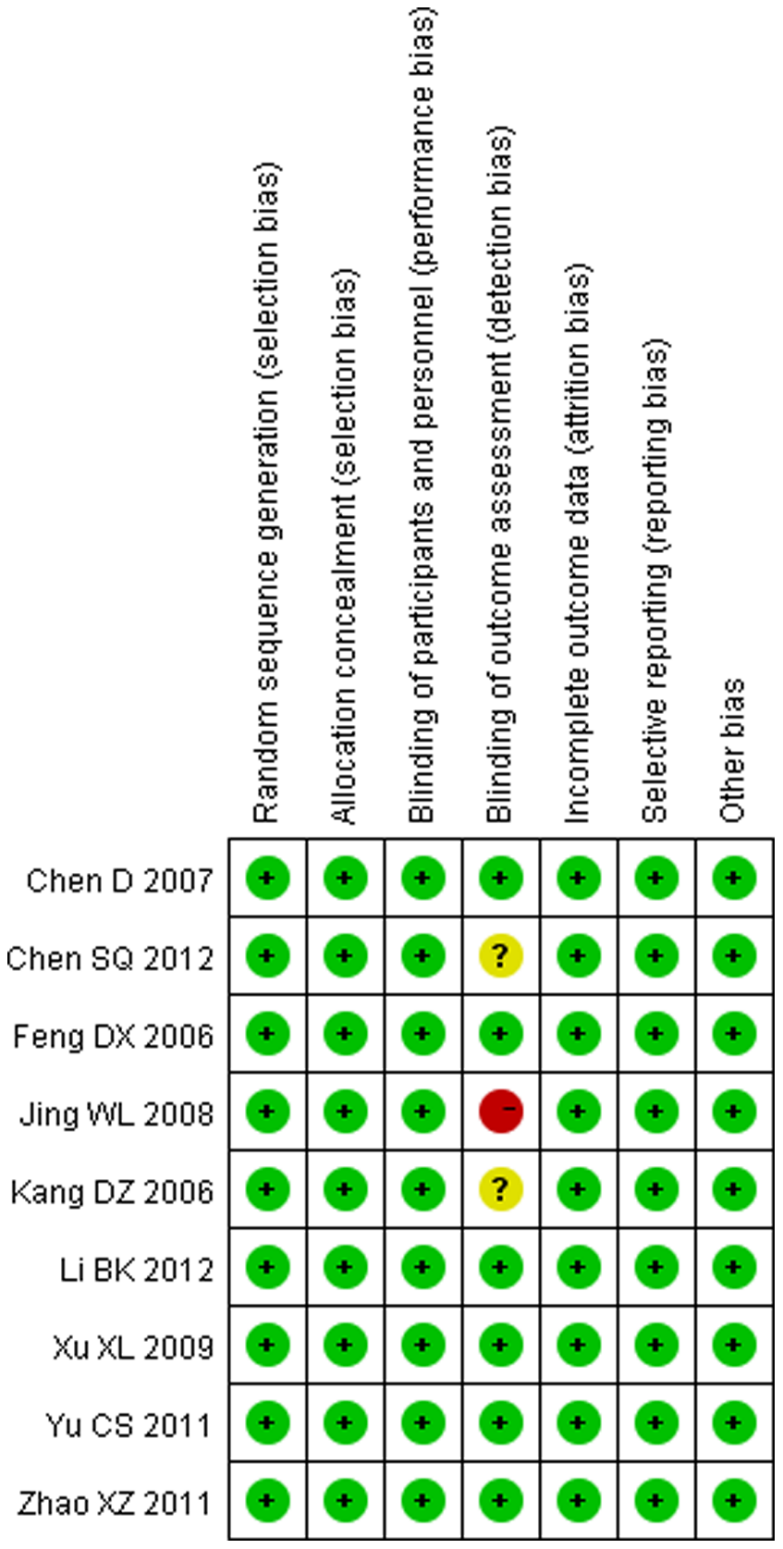
Risk of bias summary. A review of the author's judgments about each risk of bias item for each included study. + is “low risk”, − is “high risk”, ? is “unclear”.

A summary of methodological domain assessment for each study is detailed in figure 1. Only 2 studies mentioned the blinding of outcome assessment clearly. There was no blinding of outcome assessment in only 1 studies. Overall, the risk of bias was considered as a low level. (Figure 2)

### 3. BBB score in subgroups of different models

#### 3.1. BBB score at 1 week after transplantation

**Figure 3:**
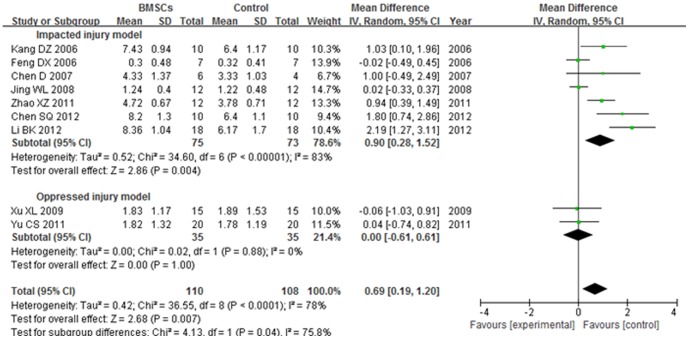
Forest plot of the differences in the BBB score of the BMSCs and control groups in different model subgroups at 1 week after transplantation. The BBB score of impacted injury model subgroup was significantly increased in the BMSCs group than the control group at 1 week after BMSCs transplantation (WMD = 0.90; 95% CI, 0.28–1.52; P = 0.004).

The BBB score of impacted injury model subgroup was significantly increased in the BMSCs group than the control group at 1 week after BMSCs transplantation (WMD = 0.90; 95% CI, 0.28–1.52; P = 0.004). The heterogeneity was high (I^2^ = 83%). No significant difference was found between the BMSCs and control groups in the BBB score of oppressed injury model subgroup (P = 1.00). No heterogeneity was found (I^2^ = 0%).The overall BBB score was significantly increased in the BMSCs group than the control group at 1 week after BMSCs transplantation (WMD = 0.69; 95% CI, 0.19–1.20; P = 0.007). The total heterogeneity was high (I^2^ = 78%). The heterogeneity between subgroups was high (I^2^ = 75.8%). (Figure 3)

#### 3.2. BBB score at 3 weeks after transplantation

**Figure 4:**
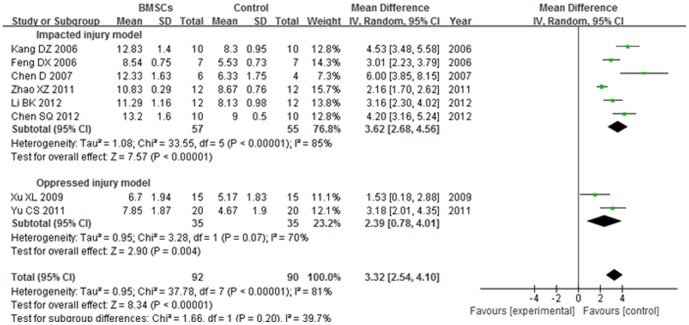
Forest plot of the differences in the BBB score of the BMSCs and control groups in different model subgroups at 3 weeks after transplantation. The BBB score of impacted injury model subgroup was significantly increased in the BMSCs group than the control group at 3 weeks after BMSCs transplantation (WMD = 3.62; 95% CI, 2.68–4.56; P<0.00001).

The BBB score of impacted injury model subgroup was significantly increased in the BMSCs group than the control group at 3 weeks after BMSCs transplantation (WMD = 3.62; 95% CI, 2.68–4.56; P<0.00001). The heterogeneity was high (I^2^ = 85%). The BBB score of oppressed injury model subgroup was significantly increased in the BMSCs group than the control group at 3 weeks after BMSCs transplantation (WMD = 2.39; 95% CI, 0.78–4.01; P = 0.004). The heterogeneity was medium (I^2^ = 70%). The overall BBB score was significantly increased in the BMSCs group than the control group 3 weeks after BMSCs transplantation (WMD = 3.32; 95% CI, 2.54–4.10; P<0.00001). The total heterogeneity was high (I^2^ = 81%). The heterogeneity between subgroups was low (I^2^ = 39.7%). (Figure 4).

#### 3.3. BBB score at over 5 weeks after transplantation

**Figure 5:**
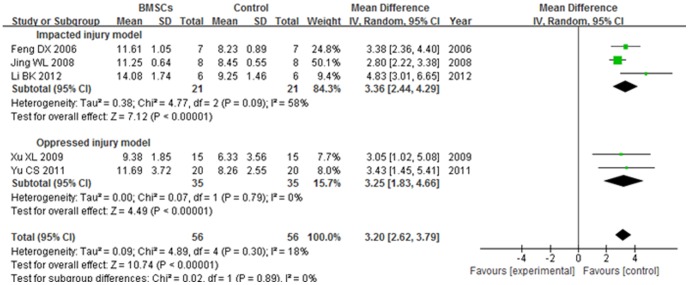
Forest plot of the differences in the BBB score of the BMSCs and control groups in different model subgroups at over 5 weeks after transplantation. The BBB score of impacted injury model subgroup was significantly increased in the BMSCs group than the control group at over 5 weeks after BMSCs transplantation (WMD = 3.36; 95% CI, 2.44–4.29; P<0.00001).

The BBB score of impacted injury model subgroup was significantly increased in the BMSCs group than the control group at over 5 weeks after BMSCs transplantation (WMD = 3.36; 95% CI, 2.44–4.29; P<0.00001). The heterogeneity was medium (I^2^ = 58%). The BBB score of oppressed injury model subgroup was significantly increased in the BMSCs group than the control group at over 5 weeks after BMSCs transplantation (WMD = 3.25; 95% CI, 1.83–4.66; P<0.00001). No heterogeneity was found (I^2^ = 0%). The overall BBB score was significantly increased in the BMSCs group than the control group at over 5 weeks after BMSCs transplantation (WMD = 3.20; 95% CI, 2.62–3.79; P<0.00001). The total heterogeneity was not found (I^2^ = 18%). The heterogeneity between subgroups was not found (I^2^ = 0%). (Figure 5).

### 4 BBB score in subgroups of different transplantation time

All studies were divided into 2 subgroups, one subgroup transplanted BMSCs at 3 days after SCI (3 d subgroup) and the other transplanted BMSCs at 7 days (7 d subgroup) after SCI.

#### 4.1. BBB score at 1 week after transplantation

**Figure 6:**
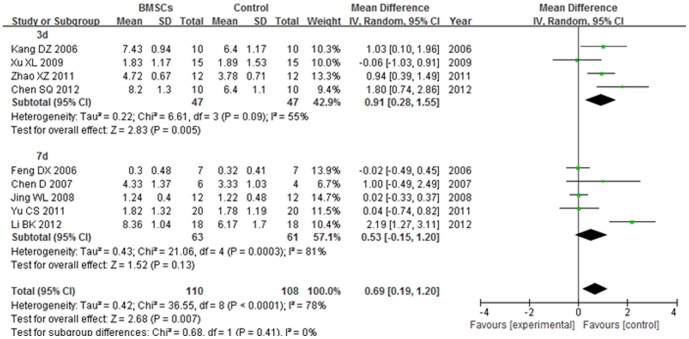
Forest plot of the differences in the BBB score of the BMSCs and control groups in different time subgroups at 1 week after transplantation. The BBB score of 3(WMD = 0.91; 95% CI, 0.28–1.55; P = 0.005).

The BBB score of 3 d subgroup was significantly increased in the BMSCs group than the control group at 1 week after BMSCs transplantation (WMD = 0.91; 95% CI, 0.28–1.55; P = 0.005). The heterogeneity was medium (I^2^ = 55%). No significant difference was found between the BMSCs and control groups in the BBB score of 7 d subgroup (P = 0.13). The heterogeneity was high (I^2^ = 81%). The overall BBB score was significantly increased in the BMSCs group than the control group at 1 week after BMSCs transplantation (WMD = 0.69; 95% CI, 0.19–1.20; P = 0.007). The total heterogeneity was high (I^2^ = 78%). The heterogeneity between subgroups was not found (I^2^ = 0%). (Figure 6).

#### 4.2. BBB score at 3 weeks after transplantation

**Figure 7:**
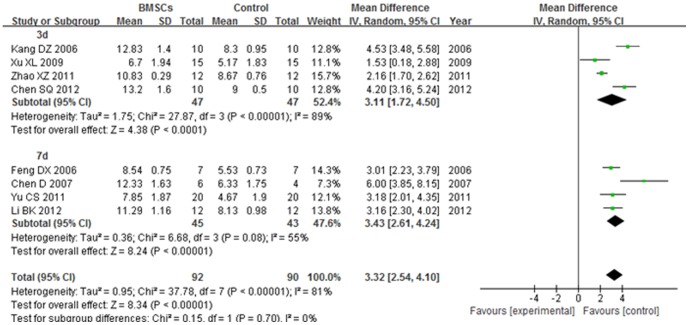
Forest plot of the differences in the BBB score of the BMSCs and control groups in different time subgroups at 3 weeks after transplantation. The BBB score of 3(WMD = 3.11; 95% CI, 1.72–4.50; P<0.0001). The BBB score of 7 d subgroup was significantly increased in the BMSCs group than the control group at 3 weeks after BMSCs transplantation (WMD = 3.43; 95% CI, 2.61–4.24; P<0.00001). The overall BBB score was significantly increased in the BMSCs group than the control group at 3 weeks after BMSCs transplantation (WMD = 3.32; 95% CI, 2.54–4.10; P<0.00001).

The BBB score of 3 d subgroup was significantly increased in the BMSCs group than the control group at 3 weeks after BMSCs transplantation (WMD = 3.11; 95% CI, 1.72–4.50; P<0.0001). The heterogeneity was high (I^2^ = 89%). The BBB score of 7 d subgroup was significantly increased in the BMSCs group than the control group at 3 weeks after BMSCs transplantation (WMD = 3.43; 95% CI, 2.61–4.24; P<0.00001). The heterogeneity was medium (I^2^ = 55%). The overall BBB score was significantly increased in the BMSCs group than the control group at 3 weeks after BMSCs transplantation (WMD = 3.32; 95% CI, 2.54–4.10; P<0.00001). The total heterogeneity was high (I^2^ = 81%). The heterogeneity between subgroups was not found (I^2^ = 0). (Figure 7).

#### 4.3. BBB score at over 5 weeks after transplantation

**Figure 8:**
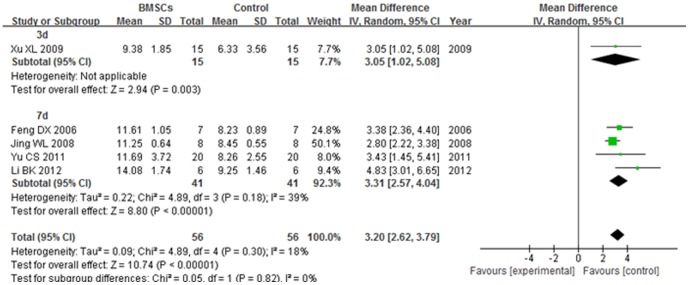
Forest plot of the differences in the BBB score of the BMSCs and control groups in different time subgroups at over 5 weeks after transplantation. The BBB score of 3(WMD = 3.05; 95% CI, 1.02–5.08; P = 0.004). The BBB score of 7 d subgroup was significantly increased in the BMSCs group than the control group at over 5 weeks after BMSCs transplantation (WMD = 3.31; 95% CI, 2.57–4.04; P<0.00001). The overall BBB score was significantly increased in the BMSCs group than the control group at over 5 weeks after BMSCs transplantation (WMD = 3.20; 95% CI, 2.62–3.79; P<0.00001).

The BBB score of 3 d subgroup was significantly increased in the BMSCs group than the control group at over 5 weeks after BMSCs transplantation (WMD = 3.05; 95% CI, 1.02–5.08; P = 0.004). The BBB score of 7 d subgroup was significantly increased in the BMSCs group than the control group at over 5 weeks after BMSCs transplantation (WMD = 3.31; 95% CI, 2.57–4.04; P<0.00001). The heterogeneity was low (I^2^ = 39%). The overall BBB score was significantly increased in the BMSCs group than the control group at over 5 weeks after BMSCs transplantation (WMD = 3.20; 95% CI, 2.62–3.79; P<0.00001). The total heterogeneity was not found (I^2^ = 18%). The heterogeneity between subgroups was not found (I^2^ = 0). (Figure 8)

## Discussion

It is proved that BMSCs are able to differentiate to neuron and neurogliocyte in vitro and vivo [20]–[22]. Compared with embryonic stem cells, olfactory ensheathing cells and Schwann cells, BMSCs have lower immunogenicity and wide source, meanwhile they are easier to extract, isolate, purify and culture [23]–[24]. BMSCs have been transplanted to cure the spinal cord injury by many scholars, at home or abroad. Although local injection to the injury position is used in most researches [25]–[27], it is hard to operate and easy to cause secondary damage. Because of the advantages, such as convenient, safe and allowed to inject for transplantation repeatedly, more and more research on intravenous transplantation of BMSCs in animals are researched. BBB score is a generally accepted method of evaluation for the degree of SCI and treatment effect. It is useful for clinical application in the future with this Meta-analysis of random animal trials.

Overall, the BBB score of BMSCs group was significantly higher than control group at 1 week, 3 weeks and over 5 weeks after transplantation. This result shows clearly that intravenous transplantation with BMSCs promotes the motor function of spinal cord injured rats. We found that there was no significant difference between BMSCs and control groups 1 week after transplantation in the subgroup of oppressed injury model. The reason may be the damage oppressed by the hemoclip was so serious that it took more time for recovery.

In consideration of the BBB rating scale without defining the locomotor recovery spontaneous or not [28]–[29], some researchers believe that if the BBB score are less than 8 point, it is completely or it includes the spontaneous recovery at least. Because they found that all rats with T10 dorsal hemisection, T10 transection, T10segment resection got about 8 in BBB scales [30]. In this paper, we find that the BBB scores are about 8 points in rats of control groups at 3 weeks and 5 weeks after spinal cord injury. In the other side, the BBB scores of transplantation groups are 2–4 points higher than the parallel control groups. Therefore, intravenous transplantation with BMSCs benefits the locomotor recovery.

The heterogeneity between impacted injury model and oppressed injury model subgroups decreased with the passage of time. It decreased from 75.8% at 1 week after transplantation to 39.7% at 3 weeks after transplantation, finally 0%. It implies that the degree of SCI and the recovery speed after transplantation in different models was different. While the heterogeneity between 3 d and 7 d subgroups was 0% at 1 week after transplantation, and the same at 3 weeks and over 5 weeks after transplantation. It means that the effect of transplantation with BMSCs at 3 days after SCI is the same as 7 days after SCI. So the inference is that the therapeutic window of BMSCs transplantation is wide. The patients of SCI may be cured with BMSCs transplantation in 7 days.

## Conclusion

Our Meta-analysis indicated that the intravenous transplantation with BMSCs is an effective therapy for SCI in rat. The motor function was increased after intravenous transplantation with BMSCs. Even at 7 days after SCI, this therapy can still work and get good effect. It must be pointed out that the BBB score is subjective partly and this result is only a report from 9 studies. Because of the limit of the rare studies at present, we just compared the effect between intravenous transplantation and control groups. We think intravenous transplantation with BMSCs is a new way of SCI treatment for patients who are intolerable or unwilling of operation.

## Supporting Information

Checklist S1 **PRISMA Checklist.**
(DOC)
